# Biologically Inspired Surgical Needle Steering: Technology and Application of the Programmable Bevel-Tip Needle

**DOI:** 10.3390/biomimetics5040068

**Published:** 2020-12-16

**Authors:** Eloise Matheson, Ferdinando Rodriguez y Baena

**Affiliations:** Mechatronics in Medicine Lab, Imperial College London, London SW7 2BU, UK; e.matheson@imperial.ac.uk

**Keywords:** cyclic control, bioinspiration, needle steering, bevel tip, PBN

## Abstract

Percutaneous interventions via minimally invasive surgical systems can provide patients with better outcomes and faster recovery times than open surgeries. Accurate needle insertions are vital for successful procedures, and actively steered needles can increase system precision. Here, we describe how biology inspired the design of a novel Programmable Bevel-Tip Needle (PBN), mimicking the mechanics and control methods of certain insects ovipositors. Following an overview of our unique research and development journey, this paper explores our latest, biomimetic control of PBNs and its application to neurosurgery, which we validate within a simulated environment. Three modalities are presented, namely a Direct Push Controller, a Cyclic Actuation Controller, and a newly developed Hybrid Controller, which have been integrated into a surgical visual interface. The results of open loop, expert human-in-the-loop and a non-expert user study show that the Hybrid Controller is the best choice when considering system performance and the ability to lesson strain on the surrounding tissue which we hypothesis will result in less damage along the insertion tract. Over representative trajectories for neurosurgery using a Hybrid Controller, an expert user could reach a target along a 3D path with an accuracy of 0.70±0.69 mm, and non-expert users 0.97±0.72 mm, both clinically viable results and equivalent or better than the state-of-the-art actively steered needles over 3D paths. This paper showcases a successful example of a biologically inspired, actively steered needle, which has been integrated within a clinical interface and designed for seamless integration into the neurosurgical workflow.

## 1. Introduction

The optimal low level control of needles for the application of percutaneous interventions [1] for Minimally Invasive Surgery (MIS) is an open research topic. Whilst passive needles have more simple kinematics compared to active needles and so are easier to model and control, active needles are better able to adapt to varying environmental conditions intraoperatively, leading to higher accuracy of path following and final placement. They can also follow trajectories along curvilinear 3D paths, which is particularly important when navigating through complex geometry [2].

The first, and earliest, category of steerable needles, consists of the base manipulation of straight, flexible needles through solid tissue, whereby the base manipulation of the robot, and the needle–tissue reaction forces, are used to move the needle along a curved path. Often, these needles have bevelled and/or precurved needle tips [3,4,5], to increase the achievable curvature. This technique is commonly used in practice and is accurate for shallow insertions, however; for deeper insertions, steerability is limited, allowing little room for adjustment and area avoidance. If the tip is not accurately placed, a reinsertion may be required, increasing the tissue trauma to the patient [6,7].

A second category includes needles with straight, stiff shafts or flexible shafts equipped with an articulated tip-mounted tool. Articulation of the tip is provided either by pivoting the straight shaft about a fulcrum located at the insertion point into the patient, introducing further tissue deformation, or by transferring the applied force to the tip with a transmission mechanism [8]. An example is the tendon driven actuated tip placed on a flexible shaft [9]. Hand held and robotic devices, both in research [10] and available commercially [11], are often used in MIS to access body cavities such as the abdomen. Work has been carried out for MRI capability with a tendon driven needle [8] to ensure accurate position sensing for intraoperative navigation control. Tip steering has the advantage of a follow-the-leader trajectory, where the shaft follows the path of the tip, and steerability is largely unaffected by the insertion depth. However, the needle–tissue interaction forces are difficult to model and predict, and nonholonomic constraints complicate the control of such designs.

**Figure 1:**
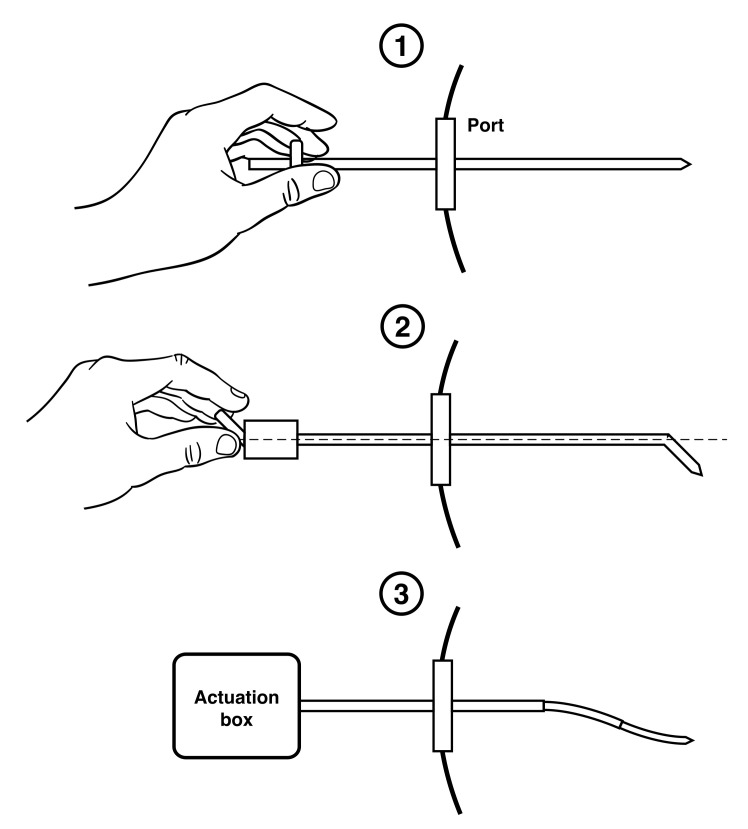
Examples of needle designs in: Category (1) manual base manipulated needle; Category (2) manual base manipulated articulated bevel-tip needle; and Category (3) active concentric tube needle.

To minimise tissue trauma, and allow for more complicated path following, a third category of needles is developing, broadly focusing on steerable, elongated and active devices. An example of a needle design in each of the third categories is shown in Figure 1. Multiple design solutions exist for this category and have been extensively reviewed in [12,13,14,15], including telescopic mechanisms such as the concentric combination of precurved elastic tubes presented in [16,17], and snake-like solutions via both shape memory alloys [18,19,20] and tendon actuation methods [21,22]. These designs also have their limitations, for example telescopic mechanisms require a priori designs, limiting the variability of the final trajectory in tissue. Shape memory alloy prototypes exhibit problems with heat dissipation, whilst tendon driven designs are problematic to miniaturise.

Commonly, designs in this category employ bevel-tip needles. These can be actively steered by using duty-cycling control techniques, whereby the rotation of the needle along its longitudinal axis allows the needle to be steered in the desired direction [5,23]. Such needles are able to follow approximately straight paths as well as curved paths in 3D, although changing the plane of steering can introduce further tissue deformation. Continuous rotation of the needle is required to achieve a straight path, and it is possible helical motions of the needle occur—leading to a small blending effect on the surrounding tissue. There also exists actively steering cannula and stylet designs, such as the one presented in [24]. The amount of curvature of the needle is dependent on offset of the bevel tip from the cannula, which is controlled by extending and retracting the stylet. To change from one plane of steering to the other, the bevel tip can be fully retracted into the cannula before being rotated, limiting the amount of tissue damage during such a manoeuvre. Normal duty-cycling methods must be applied when following straight trajectories, as a zero offset configuration approximates a normal bevel tipped needle.

Our bio-inspired Programmable Bevel-tip Needle (PBN) is in the third category. PBNs are inspired by insects that are able to use their slender ovipositors or mouthparts to both drill and steer through harder substrates, such as wood and bodies of other insects [25]. In [26], the authors used 2D and 3D motion analysis to see the motion of the fruit-fly parasitoid *Diachasmimorpha longicaudata (Braconidae)* three-valve ovipositor. They saw that the insect could steer their ovipositors in any direction relative to the body axis-with direct pushing motions in soft substrates and via reciprocal motions in stiffer substrates. This reciprocal, or cyclic, motion can reduce the net force of the ovipositor on the surrounding substrate, lowering the risk of buckling. This control motion has inspired the control of PBNs with cyclic motion, in order to reduce the deformation and strain on the surrounding tissue, with a likely reduction in damage along the needle insertion track.

PBN designs consist of at least three interlocking, bevel-tipped segments which axially slide relative to one another to achieve 3D steering. The relative offset of the segments formed at the tip creates a bending moment when inserted into soft tissue, and is related to the resulting curvature of the needle. The multi-segment design of this needle is advantageous over other single segment needles as it can lend itself to more advanced actuation methods, hence different motion profiles, and does not suffer from the constraint of following helical paths as standard bevel-tip flexible needles do. Motion profiles can be optimised towards specific parameters, e.g., to minimise tissue deformation. Such needles can be formed by collections of nitinol wires as in [27], or via interlinking plastic segments such as the PBN used in this work.

This paper presents research on the underlying motion profiles for PBNs that can be controlled in order to reduce the strain on the surrounding tissue, which could reduce the subsequent tissue damage that occurs along the needle tract—our clinical goal for this specific research. This can be achieved by employing cyclic motion, where each segment moves forward and backwards during a profile, in a similar manner to how some wasps can drill and steer their ovipositors through a harder substrate, such as bark, without buckling. A novel Hybrid Controller is presented and compared against a Direct Push controller and a fully Cyclic Controller. All three control methods have been integrated into the visual interface the surgeon would use to perform neurosurgery, where the navigation commands are measured by a control joystick. This is the first time the Cyclic and Hybrid Controllers have been tested using the surgical interface.

Section 2 tells the story of the background research and development undertaken over the last decade to produce the current design of the PBN. Section 3 describes the control methods, their design, and the system setup. Section 4 describes the experiments and results designed to test the controller performance and measure the integration success of the controllers within the surgical interface. Two experiments were undertaken by an expert user, and one experiment involved a user studies trial. Section 5 discusses these results, highlighting the areas still to be improved for the controller design and interface integration. Section 6 concludes this work and presents the upcoming trials to evaluate the full clinical setup.

## 2. Related Work

PBNs have evolved over a 13-year journey that found its roots in a blue-sky collaboration with Bath university and Prof Julian Vincent, a world leading biomimeticist and the source of inspiration of this unique design. Thanks to national and international funding, the concept matured from early proof-of-concept to a medical-grade, pre-production prototype suitable for live use, which is currently at the centre of a large-scale European consortium effort on precision neurosurgery.

**Figure 2:**
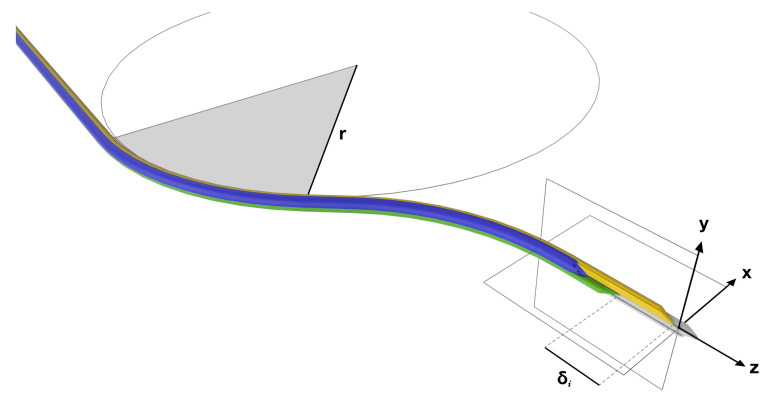
Four-segment PBN showing the radius of curvature, r, an offset between two segments, $\delta_{i}$, and the catheter tip frame.

The Enhanced Delivery Ecosystem for Neurosurgery (EDEN2020) project is a European Union Horizon 2020 Research and Innovation Action (RIA) involving six universities and two companies, which aims to develop the gold standard for one-stop diagnosis and minimally invasive treatment in neurosurgery. Imperial College has predominately been in charge of developing the needle and robotic platform, with specialist manufacturing support provided by Xograph Technologies Ltd. (Xograph, Stroud, United Kingdom). The PBN design consists of four interlocking, bevel-tipped segments that axially slide relative to one another, as shown in Figure 2. An offset of $\delta_{i}$ causes a deflection in the [x,y] frame of the needle, as it is inserted along the *z* axis. The resulting curvature can be determined by measuring the radius of curvature, R.

In [28], Ko et al. first modelled a 12 mm outer diameter PBN in 2D and experimentally calibrated the curvature and offset relationship with open (feed-forward) control. Following this, simulated 2D needle insertions were controlled with closed (feed-backward) control using the chained form representation, originally developed to control nonholonomic non-linear robotic cars. This work found that the needle curvature was approximately proportional to the steering offset. Follow up in vitro experiments are presented in [29]. Using the same needle, kinematic model and controller, experimental results demonstrate 2D trajectory following with 0.68 mm tracking error and 1.45 mm standard deviation. 

This control method was limited in regards to the magnitude of the control gains and a large initial position perturbation, which caused instability due to a number of control input constraints. In the work presented in [30] a Model Predictive Controller (MPC) considered the input and output constraints arising from the mechanism of the needle, specifically considering the maximum achievable curvature and rate of change of curvature of the needle. The tracking error model was modified such that the non-linear kinematic model of the needle was linearised, and the model was used to convert the optimisation problem into a well known quadratic programming (QP) problem, with input and output constraints represented as inequalities. These works only dealt with the needle considering 2D or planar insertions.

Further design work focused on minimising the outer diameter of the PBN, to make it closer to clinically viable and to see the effects of smaller diameter needles on the system performance. Open loop 3D steering of the needle with an outside diameter of 8 mm was presented by Burrows et al. [31]. Results from fourteen insertions into a phantom gelatin with constant offsets in eight principle axes found a linear relationship between the segment offset and needle curvature, in agreement with results previously presented for the larger size needle. In [32], a prototype with a 4 mm outer diameter was presented that was able to steer around bends with a radius of curvature of approximately 70 mm. A path planner was developed to comply with the mechanical constraints of the design to avoid needle buckling whilst avoiding obstacles. This used an iterative based gradient approach to produce smooth paths with bounded curvature gradients. Experimental results for 2D insertions into phantom gelatin using the same MPC controller as in [30] provided 0.1 mm average tracking error, with 0.64 mm standard deviation.

However, the path planner in [32] was computationally expensive. Whilst appropriate for preoperational path planning, it was limited in its usefulness for online path planning during operations. In [33] a Deformation-as-control (DAC) path planner was developed, considering a bounded curve derivative, giving a non-linear formulation which was then linearised. Using the same MPC controller from [30], experimental results using the extended DAC planner demonstrated that the system could successfully guide the needle in a 2D plane to a moving target with a total mean end position error of 0.27 mm and total mean approach angle error of $0.8^{\circ }$.

In [34], further experimentation with the same needle as in [33] was undertaken in a series of in vitro scenarios, for both single and double planar target locations. Feedback via a laser based 3D visioning system allowed closed loop control, using the same MPC controller as in [30]. By experimental calibration, a linear relationship between offset and curvature was found, as was the case in all previous experiments. These experiments demonstrated the the needle was able to steer in situ to compensate for moving targets due to soft tissue deformation. Overall, the mean positional error to reach the target was 0.46 mm with a standard deviation of 0.17 mm and the overall mean approach angle error for target orientation was $1.05^{\circ }$, with a standard deviation of $0.32^{\circ }$. These results are similar to those obtained in earlier experiments in phantom gelatin, demonstrating that the path planner and control can be successfully used to guide the needle to multiple moving targets in a plane, whilst taking into account the path constraints.

To better understand the deformation caused by the needle on surrounding tissue, Oldfield et al. [35] demonstrated a laser-based digital image correlation technique (DIC) to observe the tissue displacement around a needle as it is inserted into a transparent phantom. Building on this observation technique, it was then shown that cyclic actuation of the individual needle segments could reduce localised target motion and surrounding tissue displacement caused by the needle–tissue interaction. This was shown via FEA simulations and then in open loop gelatin phantom tissue insertions, all in 2D [36]. Specifically, cyclic motion with pullback of 30% and a 4 mm stroke length resulted in the least tissue deformation.

**Figure 3:**
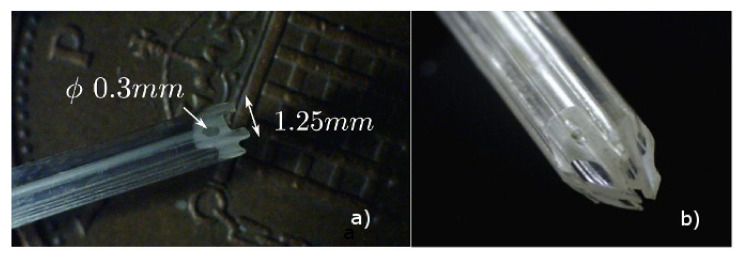
(**a**) Medical grade PBN needle cross-section; and (**b**) four-part needle PBN assembled.

After five years of research and development, the PBN design was well understood and tested in 2D. Via extrusion of a medical-grade polymer, a clinically viable sized needle with a 2.5 mm outer diameter, as shown in Figure 3, was achieved by Xograph. However, for accurate control, a model of the system in 3D was required. To expand the system to be able to handle 3D paths, initial kinematic modeling based on an UAV with fixed wings was first developed and presented by Secoli and Rodriguez y Baena [37]. However, this model could not easily account for the material properties of the extruded needle, so a different avenue, namely 3D modelling via a multi-beam approach based on Euler–Bernoulli beam theory, was explored. Finite element simulations for known loads were used to validate the multi-beam deflection model in [38], and the maximum achievable curvature was found to be 0.0192 mm±0.0014 mm${}^{-1}$ from a series of phantom trials in gelatin. These trials helped to formulate the forward model of the needle, in order to calculate the expected curvature from the known offset configuration. However, for closed loop control, it was necessary to formulate the inverse model, so an operator could provide steering commands and the system could respond with the appropriate offset configuration to achieve these.

The inverse model—determining the required offset to generate a desired curvature—was the subject of work presented in [39]. This used an optimisation algorithm to find a numerical solution based on the proposed steerability measures of the steerability index (analogous to the manipulability index of serial manipulators and geometrically proportional to the area of the steerability ellipse in curvature space) and the steering condition number (a dimensionless value representing how far or close to a singularity condition the needle is). As PBN four-segment needles are over-actuated, an optimisation technique is required to find the configuration that results in the highest steering curvature with the smoothest configuration changes. Importantly, the relationship between offsets and curvature for the 2.5 mm outer diameter needle was found to be non-linear, in disagreement with previous results for the larger diameter needles, highlighting the need to use more complex models for the forward and inverse calculations.

Recent work of a path planner has used these models for the 2.5 mm outer diameter needle in order to create 3D paths that are optimised for the needle kinematics and the start and end pose constraints of the neuro anatomy. This is important, as the effectiveness on drugs (for instance, used in chemotherapy) has been linked to the precision of the infusions of the drug at the tumour site [40]. To date, the PBN is clinically viable sized, soft, steerable, and MRI compatible. It has been fully modelled and the control and path planning tools have been created in order to allow for optimised, 3D trajectories to be achieved through brain tissue. The remaining sections of this paper focus on the control modality of the PBN to move the individual segments of the PBN to the desired offset configuration.

## 3. Materials and Methods

### 3.1. Control Methods

There are two main methods to control and move the catheter segments; the first is via a cyclic motion, whereby each segment is extended forward and then backward for each period of motion control. This controller is bioinspired from how wasps can drill into bark using their ovipositors. The second method is direct motion, whereby each segment is extended forward only. These modes are further described in Section 3.2.

Previous work presented in [41] demonstrated the performance of a cyclic controller with 30% pullback for planar insertions of a flexible, medical grade PBN needle with an outer diameter of 2.5 mm. Thirty per cent pullback was chosen as this value was found to cause the least amount of tissue deformation [42] which is hypothesised to produce less tissue damage. The main limitations of this work were that the control planning window (cyclic period) of the actuation profile was 8 s, that the model of the needle assumed a linear relationship between the offset of the needle segments and the resultant curvature of the needle [28], and that the trajectories were limited to planar paths. However, it successfully showed that the tissue deformation around the needle track was significantly reduced when compared to the direct controller and that there was no significant difference in target reaching performance.

The cyclic controller was extended in [43] to handle 3D trajectories with a cyclic period of 2 s, increasing the control frequency four-fold, and presented the design for position and velocity profiles of simulated motors to drive the four needle segments in SIMULINK and MATLAB 2017b© (MathWorks Inc., Massachusetts, USA). The results from this work showed that the cyclic controller under-steered when following curvilinear paths, the effects of which were more pronounced on 3D trajectories. Future work from this paper recommended two avenues of improvement, both of which are explored in this work;

1. the human operator can correct the performance in real-time and keep the open loop cyclic controller design as is; and
2. use a combination of direct and cyclic motion profiles according to the magnitude of offset configuration change to benefit from the timely response of the direct controller and cyclic profiles from the cyclic controller.

This work seeks to validate and optimise a controller via a full system simulation in C++ of a four-segment PBN with outer diameter of 2.5 mm, which is modelled according to Watts et al. [38], that does not assume a linear relationship between offset and resulting segment curvature. To understand the optimal control profile, three control modalities are presented, namely a cyclic actuation controller (CAC), a direct push controller (DPC), and a hybrid controller (HC), which combines both the cyclic and direct control motions according to the desired offset from the higher level controller.

Experiments were undertaken for open loop curvilinear trajectories in 3D to evaluate the path following and target reaching performance of each of the control modalities, followed by a closed, human-in-the loop, single expert user study to evaluate the same metrics. Finally, a small human user trial was performed to understand if the control modality affected the user when using the neurosurgical human machine interface. The following hypotheses are made:

**Hypothesis** **1.** *The CAC will achieve significantly less performance compared to the DPC.*

**Hypothesis** **2.** *There exists a HC that achieves the same performance metrics of the DPC while still using CAC profiles more than 75% of the insertion time*

**Hypothesis** **3.** *Users will not notice the difference in the choice of underlying control modality when using the system’s visual interface.*

### 3.2. Controller Design

Controlling the catheter requires continuous adjustment of the relative offsets between the segments. A direct push controller (DPC), first described in [30], creates the desired offset by pushing the segments forward at different speeds, though the net speed of the catheter through the tissue is pre-defined and constant. When no offset is requested, the segments each move forward at the net velocity $V_{net}$; however, when an offset is requested, the relevant segments move forward at a higher velocity, $V_{fwd}^{DPC}$.

(1) $$V_{Seg}^{DPC}\left(t\right)==\left\{\begin{array}{cc} V_{net}, & \delta =0\\ V_{fwd}^{DPC}, & \delta >0 \end{array}\right.$$

The cyclic actuation controller (CAC), first described in [41], moves the catheter by simultaneously pushing and retracting the different segments, while keeping the pre-defined and constant net speed of the catheter. This motion profile reduces the strain hence displacement of the surrounding tissue [36]. In a cyclic period, each segment moves forward and backward for part of the time—the forward and retraction speeds are bounded and constant, but the period for which each is active in a cycle is variable depending on the desired offset to be achieved. This implementation is limited in that a new command is only able to be processed once a cyclic period has finished, placing an upper bound on the control frequency, and that it takes longer to achieve the desired offset compared to the DPC [43]. However, as the net speed is constant, the insertion takes the same overall time as for the DPC on the same path.

The main equations governing the motion of the CAC are as follows. The velocity profile of one segment consists of a period of the segment extending at $V_{fwd}^{CAC}$ for $T_{fwd}^{CAC}$ seconds and a period retracting at $V_{ret}^{CAC}$ for $T_{ret}^{CAC}$ seconds. This gives the following definition for the velocity profile of one segment $V_{Seg}$ over a full actuation cycle $T_{c}$ seconds:

(2) $$V_{Seg}^{CAC}\left(t\right)=\left\{\begin{array}{cc} V_{fwd}^{CAC}, & t_{a}<t<t_{b}\\ V_{ret}^{CAC}, & t<t_{a},\;t>t_{b} \end{array}\right.$$

where $T_{fwd}^{CAC}=t_{b}-t_{a}$. In Equation (2), $t_{a}$ and $t_{b}$ define when in the cyclic period the segment extends forward, which is related to the segment order of movement that is defined a priori. To create a relative offset, a stroke modification factor, $S_{f}$, is introduced. This percentage value ranging $0\leq S_{f}\leq 1$ allows the maximum time a segment is moving forward to be increased by up to the value of the factor:

(3) $$T_{fwd_{\max}}=T_{fwd}\left(1+S_{f}\right)$$

Consequently, the retraction period of this segment will be reduced. The full mathematical model of the CAC is presented in detail in [41,43]. The control parameters used for this work are summarised in Table 1.

**Figure 4:**
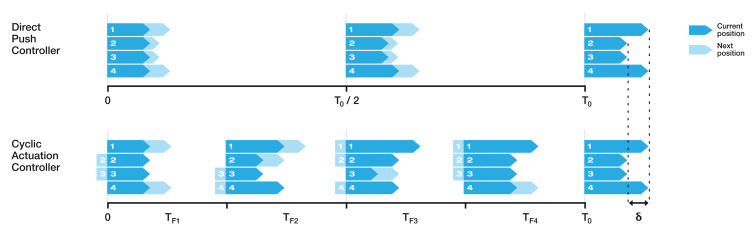
Comparison of the DAC and CAC when responding to a commanded offset, δ. $T_{o}$ refers to the time required to make the offset. $T_{F\_i}$ is the cyclic time period for which segment i moves either forward or backward.

The difference between the DPC and the CAC segment motion profiles to achieve the same offset configuration is shown in Figure 4. A hybrid controller (HC) combines the two approaches presented previously. When the desired offset, δ, is less than or equal to a predefined threshold value, $\delta_{t}$, the CPC is active. This means that the catheter will move through the tissue using the cyclic, low tissue displacement method. However, when the desired offset is higher than the threshold, the DPC is activated, achieving the desired configuration more quickly than the CPC.

(4) $$V_{Seg}^{HC}\left(t\right)=\left\{\begin{array}{cc} V_{Seg}^{CAC}\left(t\right), & \delta \leq \delta_{t}\\ V_{Seg}^{DPC}\left(t\right), & \delta >\delta_{t} \end{array}\right.$$

### 3.3. System Simulation

**Figure 5:**
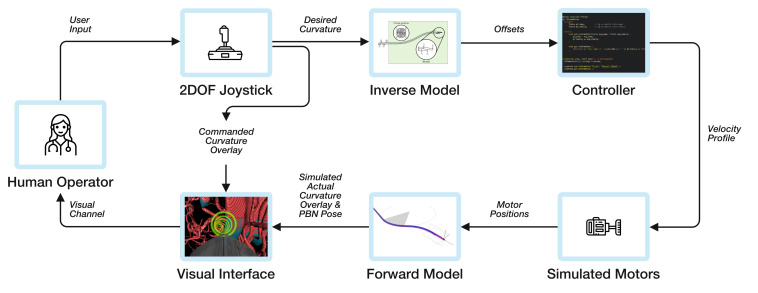
System architecture.

The Enhanced Delivery Ecosystem for Neurosurgery in 2020 (EDEN2020) project has developed a mechatronics driver for a four-segment PBN [44], where each segment is actuated by a motor connected to a linear stage that is in turn fastened to a flexible transmission line which can push or pull each segment. For this work, a full simulation of the mechatronics system in C++ has been developed in order to have a test platform for different control modalities before implementing the most optimal controller. The simulated modules have been connected with the system architecture, and communication is handled by ROS [45]. Brief descriptions of each of the modules for the system setup in simulation as depicted in Figure 5 are given below.

Actuation: The physical system uses Maxon high-precision DC brushed motors (DC16XS) with a planetary gearhead with reduction of 35:1, each equipped with a rotary magnetic encoder (1024 pulses/revolution). Each motor has a mechanical time constant of 8.57 ms, meaning it reaches 63.2% of its maximum no load speed of 10,000 rpm in 8.57 ms. The motors are connected to 1 mm pitch linear stages, and, taking into account the gearing, this means it takes 8.57 ms to reach a linear speed of 3 mm/s. The DPC has a control frequency of 5 Hz, and the CAC has a control frequency of 20 Hz. For each the net speed of the catheter is 1 mm/s, and the maximum speed of any segment when moving to create an offset is 5 mm/s for the DPC and 5.5 mm/s for the CAC; thus, the response time of the motor can be considered to be very high. In simulation, each motor velocity profile has been modelled with instantaneous acceleration, and the motor position is determined via trapezoidal numerical integration of the velocity values, with a time step of 0.05 s.

Sensing: Pose measurements of the catheter are calculated on the physical system via either embedded electromagnetic (EM) or fibre Bragg grating (FBG) optical sensors. In simulation, the pose of the catheter is calculated using the forward mechanics-based model of the catheter as detailed in [38]. This model estimates the resulting curvature of the needle given the mechanical properties of the catheter, and the relative offset between the four segments. Gaussian noise is applied to the simulated sensor readings, with a known mean and standard deviation. The error values given by the manufacturer of a single EM Aurora sensor © (NDI Medical) is 0.7 mm in position and 0.2 degrees in rotation. These are 5DOF, in the physical system each segment is sensorised with one, and the 6DOF pose is estimated from the fused measurements. For these experiments, a standard deviation of 0.1 mm about a mean of 0 mm has been applied for position measurements, and a noise of 0.7 about a mean of 0 degrees has been applied for the rotational measurements.

**Figure 6:**
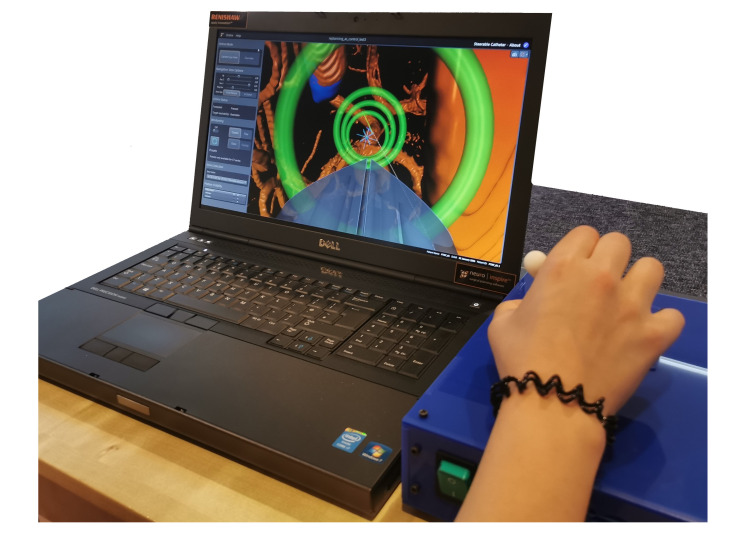
Experimental setup.

User Input: The user steers the catheter using a foot pedal for on/off motion, and a 2DOF joystick to control the curvature left/right and up/down, as shown in Figure 6, in a similar manner to the joystick of a plane.

**Figure 7:**
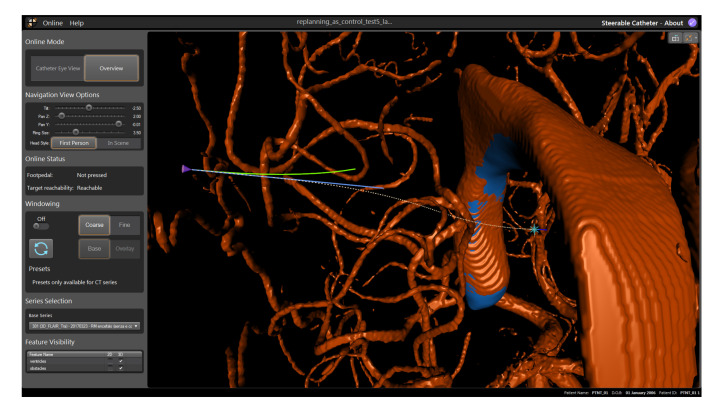
Path 2 shown with the virtual overlays in Overview Mode.

Visual Interface: This interface has been integrated into the commercial neurosurgical planning and intraoperative software neurosinpire^TM^ (Renishaw plc). The standard release of the software provides three orthogonal views of the brain—a new fourth window that visualises the 3D navigation view has now been added. In this view, the user sees a visual interface showing the preoperative MRI and CT scans of the patient, as well as a navigational “First Person View” of the catheter position in the surrounding anatomy in order to steer the catheter along the desired path. In this window, the user can also choose to see a third person “Overview View” of the environment. They are shown visual cues to help this process: the desired path; way-points as rings that they should steer through, the colour of which reflects the Euclidean error in position from the path; the expected curvature of the future catheter track as commanded by the joystick (green overlay); and the actual curvature of the future catheter track as predicted by the forward model (blue overlay). Further details of the interface are published in [46] and an image of the online mode “First Person View” is depicted in Figure 6, and the path and overlays in “Overview View” mode are shown in Figure 7.

## 4. Results

### 4.1. Experimental Design

Three experiments were setup to test the different control modalities when moving the catheter along a desired path. Ethical approval for the experiments was given by the Imperial College London Joint Research Compliance Office (ICREC 18IC4564). The paths were created using the path planner presented in [47]. Based on the Adaptive Hermite Fractal Tree (AHFT) method, the path planner generates 3D obstacle-free trajectories that satisfy curvature constraints given a specified start and target pose. The obstacle map is generated from segmented anatomy from MRI data sets of the human brain. The experimental hardware setup for all experiments consisted of a laptop showing the visual interface, the input joystick (both shown in Figure 6), and a foot pedal for on/off control.

### 4.2. Experiment 1: Open Loop Control

Two trajectories were generated: a single curve planar trajectory at maximum curvature, and a double curve 3D trajectory at maximum curvature. The maximum curvature was ρ=1/r=0.012 mm${}^{-1}$, which was experimentally achieved without buckling and corresponds to a 20 mm offset between the segments. The same open loop commands were given to each of the controller modalities to highlight the differences in performance, if any, between them. The curvatures $k_{1},k_{2}$ (${\mathrm{mm}}^{-1}$) defined in the two ortho-normal planes [x,y] planes, respectively, as in Figure 2, and radii of curvature (*r*) mm for the single curve trajectory were

$$\begin{array}{cc} \left[k_{1}\;k_{2}\right]= & \left[0.012,\;0.012\right]\\ {}[r_{Y}\;r_{Z}]= & \left[83,\;83\right] \end{array}$$

and for the double curve trajectory were

$$\begin{array}{cc} \left[k_{1}\;k_{2}\right]= & [0.012,\;0.012;\\ \; & -0.012,\;0.012]\\ {}[r_{Y}\;r_{Z}]= & [83,\;83;\\ \; & -83,\;83] \end{array}$$

**Figure 8:**
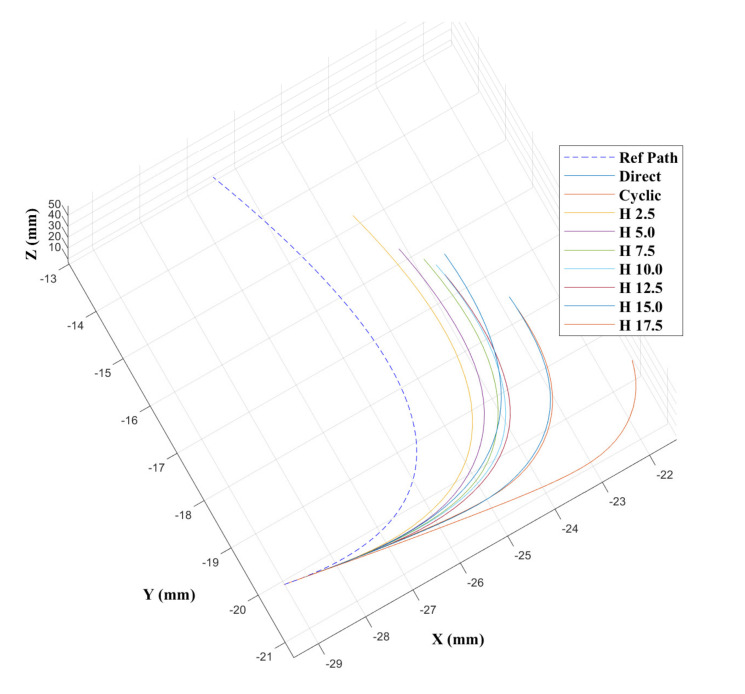
Catheter trajectories for the single bend path.

**Figure 9:**
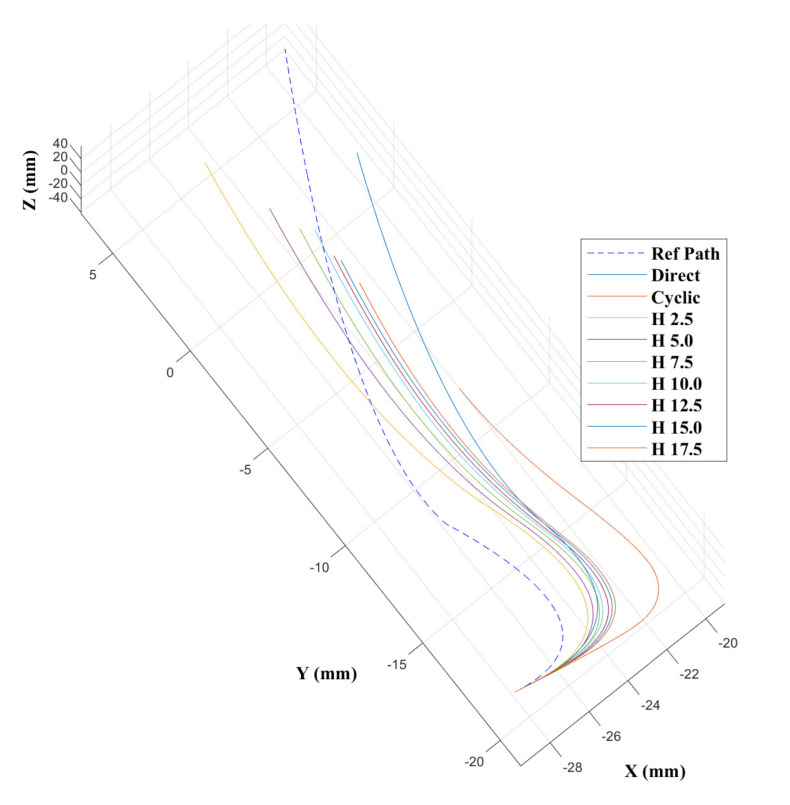
Catheter trajectories for the double bend path.

**Figure 10:**
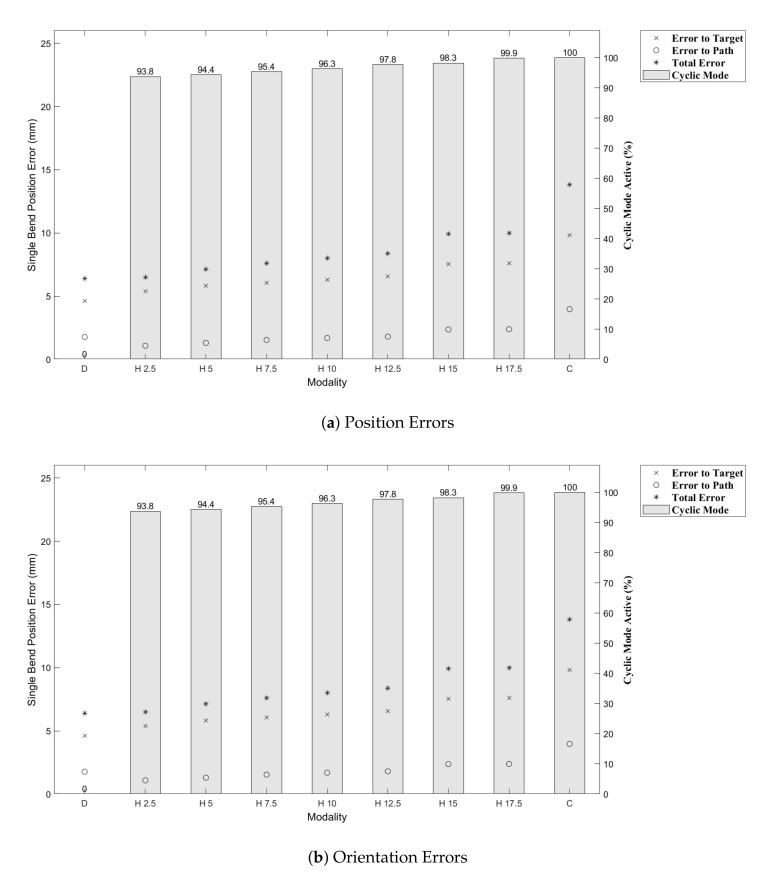
Single bend trajectory errors with per cent CAC active time shown as the bar plots.

**Figure 11:**
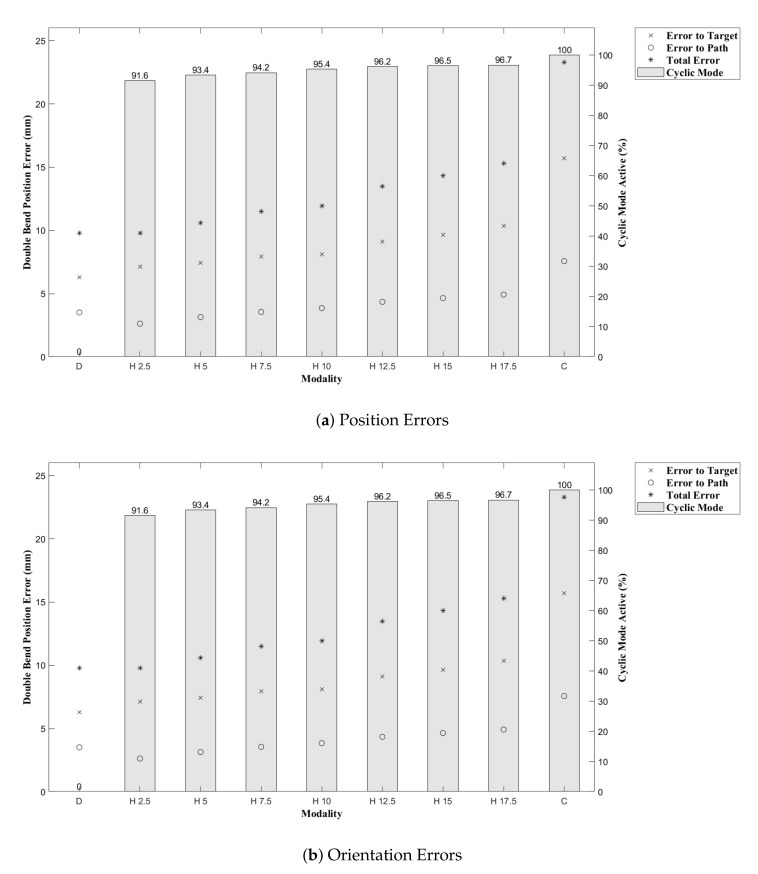
Double bend trajectory errors with per cent CAC active time shown as the bar plots.

Results from [43] show that the CAC exhibited under steering, particularly over 3D trajectories. This effect is replicated here, as can be seen from the trajectory tracks in Figure 8 and Figure 9. Indeed, we see increasing path following and target reaching errors for both the single and double bend trajectories as the HC threshold parameter increases from 0 (DPC) to 20 mm (CAC) (see Figure 10 and Figure 11).

In the author’s experience, planned trajectories through real neuro-anatomy generally only require one or two shallow curves to reach the target from the entry point, and so the per cent of time the CAC is activated for these trajectories is representative of a realistic scenario. The large control inputs requiring offset configuration changes above the threshold value normally occur when the user needs to fully change direction—which would happen at the beginning of each path and at the junction of the double bend curve. At other times, only small adjustments are necessary, meaning a HC is attractive to use as the CAC can be active for the majority of time, and the DPC can be activated only when a large offset is requested.

In Figure 10 and Figure 11, we can see there is a steady increase in path following and target reaching error for both the position and orientation for the single and double bend trajectories. Notably, the target positioning error even for the best case, the DPC, is still >5 mm, a clinically unacceptable result highlighting the need for closed loop control. The cyclic controller is active for more than 90% of the time when the HC is used, and the HC has similar performance to the DPC at low threshold values $\delta_{t}\leq 5$ mm. In the real system, there can be some misalignment of the catheter segments, ∼2 mm, during an insertion due to the elasticity of the plastic segments. For this reason, a HC threshold value of $\delta_{t}=5$ mm is chosen for Experiments 2 and 3, as this is larger than any expected misalignment with a safety factor of 2 and gives the next best results for the HC.

### 4.3. Experiment 2: Expert Single-User Closed Loop Control

Three paths were generated by the path planner in order to provide trajectories the catheter could reasonably be expected to follow during a neurosurgical procedure, each with a different target and initial insertion point into the skull, corresponding to paths approximately 60 mm in length—Path 2 in overview mode is shown in Figure 7. A single, expert user was asked to follow these curves to the best of their ability in order to reach the target. Three low level modalities were tested: the DPC, the CAC, and the HC with the threshold parameter, $\delta_{t}=5$ mm, which was chosen based on the results of Experiment 1. As the needle moves at a constant net speed of 1 mm/s, each insertion took the user approximately 1 min to complete once they had started the motion via the foot pedal.

The user made five insertions for each of the controller modalities over each path. The underlying low level control modality was changed unknowingly to the user following a Latin squares assignment. The user was asked to achieve the following objectives in decreasing order of priority.

1. Obj 1: Reach the target pose with the catheter tip
2. Obj 2: Follow the desired path

Results from [41] show that for planar insertions of a single or double bend trajectory, there was no significant difference between the target reaching or path following errors for the DPC or the CAC when a high level MPC controller was employed. However, the MPC used the linear model of the needle to simplify the model calculations and for the purpose of this paper, we use human-in-the-loop high level control, as it is not currently clinically accepted to have a fully autonomous system performing neurosurgery.

**Figure 12:**
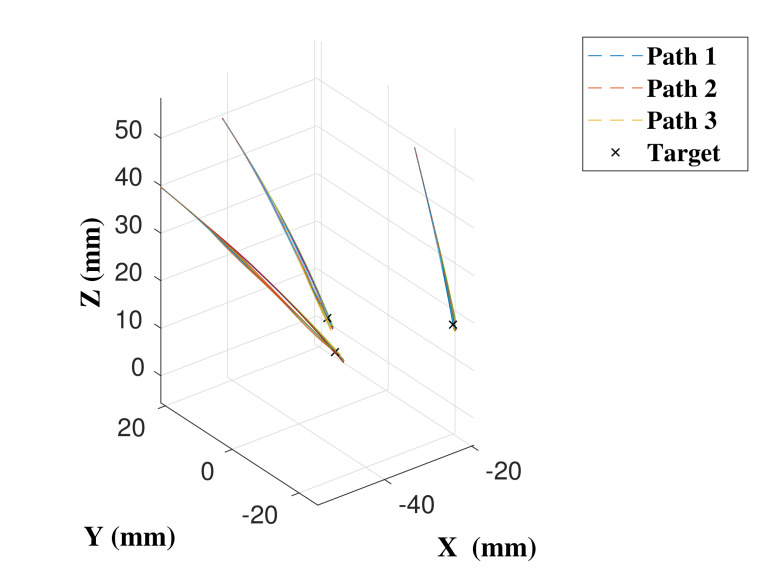
Catheter trajectories of the expert user, where each trajectory is from a different starting point in the skull to a different target located roughly centrally in the brain.

**Figure 13:**
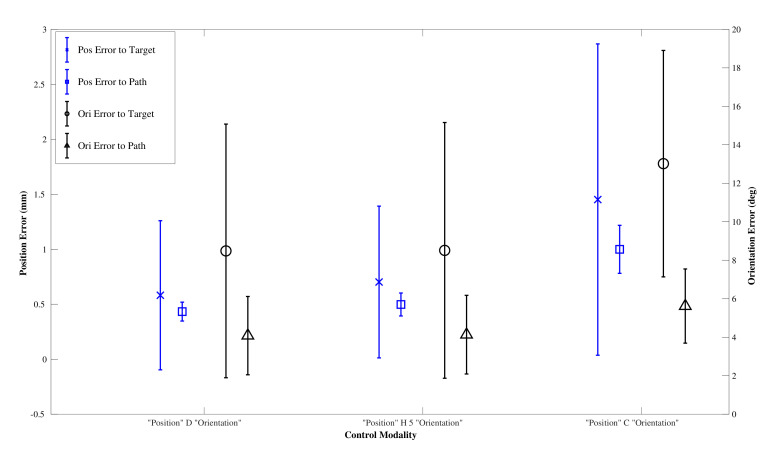
Performance results for the Expert User.

An expert user undertook five insertions for each path using the DPC, HC with threshold of $\delta_{t}=5$ mm, and the CAC, with the trajectories followed shown in Figure 12 and performance results in Figure 13, with details in Table 2.

After testing for normal distributions, a one-way between modalities ANOVA was conducted for each of the performance metrics: path following position and orientation errors and target reaching position and orientation errors. There was a significant difference for the path position error (F(2,42)=65.63,p<0.001). Post hoc comparisons using the Tukey HSD test indicated that the mean path position error for the CAC (M=0.999,SD=0.038) was significantly higher than those for the DPC (M=0.433,SD=0.038) and the HC (M=0.498,SD=0.038). Taken together, these results show the CAC displays higher path following position error compared to the DAC and the HC, but that all modalities result in similar performance in reaching the target pose. The average percentage of cyclic mode employed by the HC over all trials and all paths was 79.1±6.6%.

### 4.4. Experiment 3: Multi-User Trial

To validate the real-time performance of the visual interface and teleoperated joystick for the PBN navigation system when using different low level controller modalities, a human study (n=5) trial measured the performance of multiple users under controlled conditions. The full experiment protocol took each user approximately 1.5 h to complete. A path trajectory was generated for the training round, and the user completed five insertions using the DPC and five insertions using the CAC in order to become familiar with the system. After training, the same three trajectories as in Experiment 2 were used for the user trial.

Each user completed three insertions for each of the three modalities (DPC, HC, and CAC) over the three paths based on a Latin square assignment—giving nine insertion performance results per modality per user, or a total of 45 trials per modality. The hybrid controller threshold parameter was set to be $\delta_{t}=5$ mm as in Experiment 2, and users were asked to achieve the same objectives as in Experiment 2.

**Figure 14:**
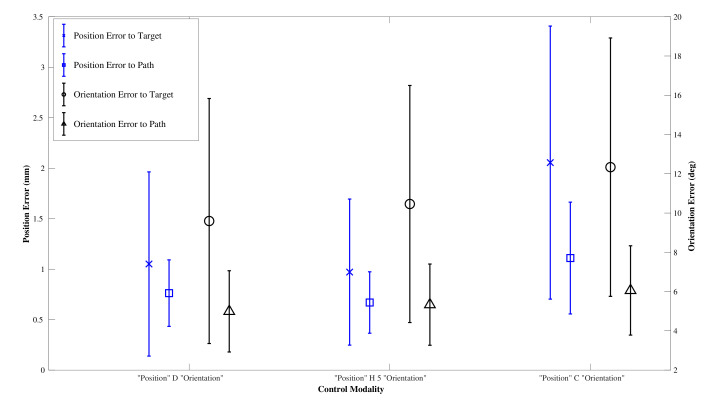
Performance results for the user trial.

The results from the user trial of non experts (n=5) are given in Figure 14 and Table 3. All users were right handed and between the ages of 25 and 35.

After testing for normal distribution, a one-way between modalities ANOVA was conducted for each of the performance metrics: path following position and orientation error and target reaching position and orientation error. There was a significant difference for the means of the target position (F(2,132)=15.48,p<0.001) and the path position error (F(2,132)=14.34,p<0.001). Post hoc comparisons using the Tukey HSD test indicated that the mean target position error for the CAC (M=2.055,SD=0.154) was significantly higher than those for the DPC (M=1.051,SD=0.154) and the HC (M=0.971,SD=0.154), and that the mean path position error for the CAC (M=1.110,SD=0.061) was significantly higher than those for the DPC (M=0.762,SD=0.061) and the HC (M=0.670,SD=0.061). Taken together, these show that the users could not compensate for the extra errors introduced by the CAC, and that the CAC modality had higher target reaching, and path following, position errors than the DPC. There was no significant difference in the performance of any metric between the DPC and the HC. The average percentage of cyclic mode employed by the HC over all trials and all paths was 77.0±10.3%.

Users were asked to complete a short survey when they finished the experiment asking which modality they found the easiest to use, and for which they thought their performance was the best as well as an open ended question to leave other comments. Sixty per cent of users did not notice a difference in difficulty between the modalities, and 40 per cent thought the DPC was the easiest to use. Eighty per cent of users did not notice a difference in performance between the DPC and the HC, and 20 per cent of users thought their performance was best when using the DPC. Comments from the users noted that ’The CAC was particularly hard to control’ and that ’They were frustrated the needle was turning to slowly when using the CAC’. These initial qualitative results show that there is little difference in user perception when using the DPC or the HC, but that the CAC has notable performance loss (agreeing with the quantitative results).

## 5. Discussion

The results of Experiment 1 highlight the trade off among under-steering, hence performance, and per cent of time the CAC is active. As the CAC can reduce tissue deformation, which is hypothesised to reduce tissue damage, it will be necessary to quantify the magnitude of this effect during in vivo trials via functional analysis of the tissue tract after the surgery. In this way, the final choice of optimal HC threshold value can be chosen.

The results of Experiments 2 and 3 indicate that the CAC has significantly lower performance than the DPC (validating H1). Here, performance is measured by our metrics of the error measurements of the needle tip to the target position and orientation and path position and orientation. The CAC had significantly higher error in path position for the expert user and in target and path position for the non-expert users. However, it should be noted that the results from Experiment 2 are only from one user, and it may be too early to draw this conclusion for expert users. In Experiment 2, the result of the CAC having significantly higher error only in the path following position when compared to the DPC or the HC was surprising. It highlights that the expert user was able to mitigate the error introduced by the under steering of the CAC to achieve the target pose as per the first objective, but possibly at the expense of the path following metric which was the second objective. The user trial in Experiment 3 shows that less expert users are not able to compensate for the CAC error in target reaching performance, hence an HC could also be attractive as less training may be required from the users to achieve expert results. We found an HC with no significant performance differences compared to the DPC when used by both an expert and a group of non-experts, with $\delta_{t}=5$ mm, who used CAC motion profiles more than 75% of the time (validating H2). From Experiment 3, H3 has not been validated—while the users did not notice a difference between the DPC and the HC control modalities, 40% noticed the performance difference of the CAC and found it harder to use. This supports the papers ultimate conclusion that the HC modality is the best choice of control for the PBN. Future work should further explore the HC parameters to see if even better performance can be achieved with different threshold values, and evaluate a bigger group of expert users.

All modes show orientation errors, with the CAC showing the worst case of target orientation/heading error of 12.34±6.58, and the DPC having 9.60±6.24. The accuracy of target heading can be linked to how users prioritise the task. As they reach the end of the path, if there is a position error, the user can turn directly to the target increasing the heading error, although lowering the position error. This is supported by the data that the path orientation error, or how well the user keeps the path heading as they track the desired path, is around half of that for the target heading error. To aid this, further instructions should be given to the users such that they prioritise heading errors more highly at the target, and visual overlays or haptic indicators could be developed in the user interface to better help them understand the magnitude of the heading error as this can be difficult to understand from the current setup.

## 6. Conclusions

Following a 10-year research and development journey, this paper summarises the main milestones associated with a novel, biologically inspired needle steering system, followed by our latest control approaches, validated in silico. These experiments highlight how cyclic motion control, which is also biologically inspired, can be delivered optimally though a blended approach, where the HC threshold value must be optimised under appropriate surgical conditions. This will be the focus of future research work for the low level control of the PBN, as well as further developing the Human–Robot Interface, both of which must be evaluated by clinicians.

The EDEN2020 system is currently being used for in vivo clinical trials with an animal (ovine) model, expected to be completed in Q1 of 2021. The trial will evaluate the safety of the system, as only a gadolinium contrast solution is being injected into healthy tissue. It will also evaluate the accuracy of path following and target reaching performance based on intraoperative sensing (FBG fibres and ultrasound real-time measurements) and from pre- and postoperative CT and MRI imaging comparisons. Upon completion, a pre-commercial prototype of the EDEN2020 platform will have been fully developed under a quality management system, and the safety data from the trial will help drive the commercialisation of the project and inform future efficacy trials.

## Figures and Tables

**Table 1:** Controller parameters.

Parameter	Symbol	Value	Unit
Cyclic Period	$T_{c}$	2	s
Cyclic Frequency	*f*	0.5	Hz
Number of segments	$Seg_{num}$	4	-
Minimum radius of curvature	*R*	70	mm
Net Velocity	$V_{net}$	1	mm/s
DAC Forward Velocity	$V_{fwd}^{DAC}$	5	mm/s
CAC Forward Velocity	$V_{fwd}^{CAC}$	5.5	mm/s
CAC Retraction Velocity	$V_{ret}^{CAC}$	0.5	mm/s
Percentage Pullback	Pullback	27	%
Maximum Stroke Factor	$S_{f}$	0.5	
Maximum Stroke Length	$S_{max}$	4.1	mm
Minimum Stroke Length	$S_{min}$	1.4	mm
Segment Period Forward	SP	0.5	s
Segment Period Forward Max	$SP_{max}$	0.75	s
Segment Period Forward Min	$SP_{min}$	0.25	s
Hybrid Threshold Value	$\delta_{t}$	2.5–17.5	mm

**Table 2:** Expert user results.

Mean Error Metric	DPC	HC	CAC
Target Position (mm)	0.58±0.68	0.70±0.69	1.45±1.41
Target Orientation (deg)	8.48±6.60	8.51±6.65	13.02±5.89
Path Position (mm)	0.43±0.09	0.50±0.10	1.00±0.22
Path Orientation (deg)	4.08±2.03	4.13±2.04	5.62±1.92

**Table 3:** User trial results.

Mean Error Metric	DPC	HC	CAC
Target Position (mm)	1.05±0.91	0.97±0.72	2.06±1.35
Target Orientation (deg)	9.60±6.24	10.46±6.04	12.34±6.58
Path Position (mm)	0.76±0.33	0.67±0.30 v	1.11±0.55
Path Orientation (deg)	4.99±2.07	5.33±2.07 v	6.06±2.28
